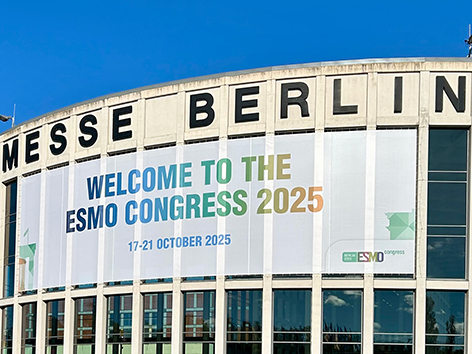# European Society for Medical Oncology (ESMO) congress 2025

**DOI:** 10.1016/j.lanepe.2025.101528

**Published:** 2025-11-02

**Authors:** Ivana Nedic

The European Society for Medical Oncology (ESMO) 2025 took place in Berlin from 17 to 21 October, bringing together oncology professionals from across the globe. The congress welcomed nearly 37,000 attendees and featured around 600 speakers, 213 scientific sessions, and close to 3,000 abstract presentations. Ivana Nedic attended the meeting and shares selected highlights from this year's programme.

## Neoadjuvant trastuzumab deruxtecan with or without chemotherapy versus standard of care for high-risk HER2-positive early breast cancer: phase III DESTINY-Breast 11 trial

Dr Nadia Harbeck (Munich, Germany) presented results from the phase 3 DESTINY-Breast 11 trial (NCT05113251), comparing neoadjuvant trastuzumab deruxtecan (T-DXd) alone or followed by paclitaxel + trastuzumab + pertuzumab (T-DXd-THP) versus dose-dense doxorubicin + cyclophosphamide followed by THP (ddAC-THP) in adults with untreated high-risk HER2-positive early breast cancer. The primary endpoint was pathological complete response (pCR), with event-free survival (EFS) and safety as secondary endpoints. Among 641 patients, pCR rates were 67.3% for T-DXd-THP and 56.3% for ddAC-THP (Δ 11.2%, 95% CI 4.0–18.3; P = 0.003), with consistent benefit in both hormone receptor–positive (61.4% vs 52.3%) and –negative (83.1% vs 67.1%) subgroups. Grade ≥3 adverse events occurred in 37.5% and 55.8% of patients, respectively; adjudicated interstitial lung disease (ILD)/pneumonitis in 4.4% vs 5.1%; and left-ventricular dysfunction in 1.9% vs 9.0%. An early trend toward improved EFS was observed (HR 0.56; 95% CI 0.26–1.17). T-DXd-THP demonstrated higher efficacy and lower toxicity than the anthracycline-based standard of care, supporting it as a potential new neoadjuvant option for high-risk HER2-positive early breast cancer.

## Safety and performance of a multi-cancer early detection (MCED) test in an intended-use population: initial results from PATHFINDER II

Dr Nima Nabavizadeh (Portland, United States) presented interim findings from the registrational PATHFINDER II study (NCT05155605) evaluating the Galleri® multi-cancer early detection (MCED) test, which analyses cell-free DNA in blood to identify cancer signals and predict their origin. The prospective, multicentre study enrolled 35,878 adults aged ≥50 years with no recent cancer diagnosis, the largest interventional MCED study conducted to date. Among 23,161 participants with 12-month follow-up, 0.93% had a positive test result. Specificity was 99.6% and positive predictive value 61.6%, with the predicted cancer signal origin correct in 92% of cases. The addition of MCED testing increased screen-detected cancers nearly seven-fold compared with standard screening alone, detecting 133 cases, 75% of which lacked existing screening options. Over half of new detections (53.5%) were diagnosed at stage I–II. These results support the test's safety and clinical utility for population-scale cancer screening.

## ctDNA-guided adjuvant atezolizumab vs placebo in muscle-invasive bladder cancer: phase III IMvigor011 trial

Prof Thomas B. Powles (London, United Kingdom) presented results from the phase III IMvigor011 trial (NCT04660344), a global randomised study to test a circulating tumour DNA (ctDNA)-guided approach to adjuvant immunotherapy in muscle-invasive bladder cancer. The study enrolled 761 patients post-cystectomy with no radiographic evidence of disease, who underwent serial ctDNA monitoring for up to one year. Among 250 ctDNA-positive patients randomised 2:1 to atezolizumab or placebo, adjuvant atezolizumab significantly improved disease-free survival (HR 0.64; 95% CI 0.47–0.87; P = 0.0047) and overall survival (HR 0.59; 95% CI 0.39–0.90; P = 0.0131) versus placebo at a median follow-up of 16.1 months. Grade 3/4 treatment-related adverse events occurred in 7.3% with atezolizumab and 3.6% with placebo, and fatal events in 1.8% and 0%, respectively. Among the 357 patients who remained ctDNA-negative throughout surveillance, recurrence risk was low (DFS 88.4% at 2 years). These data establish ctDNA-guided adjuvant immunotherapy as a feasible and effective strategy for identifying patients most likely to benefit from treatment after cystectomy.

## Bemarituzumab plus chemotherapy for advanced FGFR2b-overexpressing gastric or gastroesophageal junction cancer: phase III FORTITUDE-101 trial

Sun Young Rha (Seoul, Republic of Korea) presented results from the phase III FORTITUDE-101 trial (NCT05052801) evaluating bemarituzumab (BEMA), a first-in-class anti-FGFR2b antibody, in combination with chemotherapy for patients with FGFR2b-overexpressing, unresectable, locally advanced, or metastatic gastric or gastroesophageal junction cancer. Patients were randomized to BEMA + mFOLFOX6 or placebo + mFOLFOX6. In the FGFR2b ≥ 10% tumour cell subset, BEMA significantly improved overall survival at the primary analysis (median 17.9 vs 12.5 months; HR 0.61; 95% CI 0.43–0.86; P = 0.005) and progression-free survival (median 8.6 vs 6.7 months; HR 0.71; 95% CI 0.53–0.95; P = 0.019). Longer-term descriptive follow-up showed attenuation of the survival benefit (median OS 14.5 vs 13.2 months; HR 0.82; 95% CI 0.62–1.08). Grade ≥3 treatment-emergent adverse events were more frequent with BEMA, primarily corneal events. These results demonstrate a survival benefit with BEMA in FGFR2b-overexpressing gastric and gastroesophageal junction cancers and support further investigation in ongoing studies such as FORTITUDE-102.

## Launch of the fifth edition of the European Code Against Cancer (ECAC5)

Dr Carolina Espina (Lyon, France) presented the fifth edition of the European Code Against Cancer (ECAC5), developed by the International Agency for Research on Cancer (IARC) under the auspices of the World Health Organization and the European Commission. ECAC5 provides 14 evidence-based recommendations for cancer prevention, covering individual lifestyle choices such as tobacco and alcohol reduction, healthy diet, physical activity, and participation in organised cancer screening programmes. For the first time, the Code also introduces complementary recommendations for policymakers aimed at creating healthier environments and strengthening population-level prevention strategies. Key updates include the inclusion of lung cancer screening as a recommended programme, expansion of HPV vaccination to boys as well as girls, and recognition of ultra-processed foods as a dietary factor to limit. ECAC5 marks a shift from individual-focused prevention to a combined individual and systemic approach, reinforcing the Code's role as a cornerstone for cancer prevention policy across Europe.

**Figure:**